# Regulation of Exacerbated Immune Responses in Human Peripheral Blood Cells by Hydrolysed Egg White Proteins

**DOI:** 10.1371/journal.pone.0151813

**Published:** 2016-03-23

**Authors:** Daniel Lozano-Ojalvo, Elena Molina, Rosina López-Fandiño

**Affiliations:** Instituto de Investigación en Ciencias de la Alimentación (CIAL, CSIC-UAM), Nicolás Cabrera 9, 28049, Madrid, Spain; Institute for Virus Research, Laboratory of Infection and Prevention, JAPAN

## Abstract

The anti-allergic potential of egg white protein hydrolysates (from ovalbumin, lysozyme and ovomucoid) was evaluated as their ability to hinder cytokine and IgE production by Th2-skewed human peripheral blood mononuclear cells (PBMCs), as well as the release of pro-inflammatory factors and generation of reactive oxygen species from Th1-stimulated peripheral blood leukocytes (PBLs). The binding to IgE of egg allergic patients was determined and the peptides present in the hydrolysates were identified. The hydrolysates with alcalase down-regulated the production of Th2-biased cytokines and the secretion of IgE to the culture media of Th2-skewed PBMCs, and they significantly neutralized oxidative stress in PBLs. The hydrolysates of ovalbumin and ovomucoid with pepsin helped to re-establish the Th1/Th2 balance in Th2-biased PBMCs, while they also inhibited the release of pro-inflammatory mediators and reduced oxidative stress in PBLs treated with inflammatory stimuli. The hydrolysates with alcalase, in addition to equilibrating Th2 differentiation, exhibited a low IgE-binding. Therefore, they would elicit mild allergic reactions while retaining T cell-stimulating abilities, which might correlate with an anti-allergic benefit.

## Introduction

The self-reported prevalence of allergy to common foods in Europe ranges from 0.1% to 6% and, although these figures are above the estimates based on objective assessments, existing data indicate a high incidence of food allergy which is rapidly increasing in westernized countries [1, 2]. Most of the food-induced hypersensitivity reactions are IgE-mediated. In these cases, allergic sensitization occurs when, in susceptible individuals, usually harmless antigenic proteins enhance T lymphocyte immunogenicity and differentiation into Th2 cytokine-producing cells, what results in the activation of B lymphocytes to IgE-producing plasma cells. IgE antibodies bind to the surface of tissue mast cells and blood basophils so that, on re-exposure to the food, the allergens cross-link the cell bound specific IgE, triggering the release of mediators responsible for the allergic symptoms. Even if the immunological mechanisms are not fully understood, the differentiation and expansion of Th2 cells that secrete IL-4, IL-5 and IL-13 is considered to drive the allergic response, not only through the production of IgE, but also through the generation of chemokines that attract other cells, such as mast cells and eosinophils, and the development of the inflammation characteristic of a Th2 cytokine milieu [3].

Among the features of food proteins that play a role in their capacity to induce and allergic response, it is recognized their ability promote Th2 effector pathways, favouring Th2 rather than Th1 immunity [4]. Th1 immune activation is defined by a distinct pattern of cytokine expression, characterized by the release of IFN-γ by activated T lymphocytes, which triggers the secretion of pro-inflammatory cytokines, such as TNF-α and IL-8, and the generation of reactive oxygen species (ROS) that further accelerate inflammatory and oxidant responses, usually directed towards antimicrobial and antitumor defence mechanisms [5].

Despite the apparent opposite effects of Th2 and Th1 immunity, there are examples of antioxidant and anti-inflammatory compounds that decrease the effects mediated by IFN-γ and the intracellular pathways that accelerate the generation of ROS [6], but also reduce the expression of Th2 cytokines and IgE levels in response to food allergens in mice [7–9]. Indeed, other cell types are involved, such as Treg, which act as key immune-regulators through direct and indirect effects, including the secretion of IL-10, and play a pivotal role in the maintenance of immune tolerance and suppression of allergic inflammation [10]. Thus, polyphenols can modulate different phases of the allergic response, inhibiting the production of Th2 cytokines, while their antioxidant capacity limits the extent of cellular injury from free radicals [11–12]. Similarly, the ability of dietary omega-3 fatty acids to suppress IL-4 and IL-13 secretion by mast cells, which contributes to decrease the susceptibility to develop allergy and the severity of the symptoms, is associated with the inhibition of the generation of ROS [13–15].

The production of allergen derivatives that are hypoallergenic and immunogenic is an attractive strategy for the development of immunotherapy agents for IgE-mediated allergies. Peptide immunotherapy focuses on the properties of fragmented allergens, which contain T cell stimulating epitopes but are not capable of cross-linking IgE on basophiles and mast cells [16]. The administration of hydrolysates of egg proteins or combinations of synthetic peptides to mice sensitized against those proteins protects against anaphylaxis and reduces serum concentrations of specific IgE antibodies and histamine [17–19]. In addition, prior administration of hydrolysed egg white with low IgE-binding to mice can prevent subsequent sensitization and progress of egg allergy [20].

The results available so far suggest that, in addition to having lost their ability to induce anaphylactic reactions, certain peptides could present immunomodulatory properties, so that they could specifically drive immune responses in a certain direction. The aim of this work was to assess the potential of egg white protein hydrolysates to deviate an unbalanced immune status, such as that representative of food allergy. The immunomodulatory potential of the hydrolysates was evaluated as their ability to hinder, on the one hand, cytokine and IgE production by Th2-skewed human peripheral blood mononuclear cells (PBMCs) and, on the other hand, the release of pro-inflammatory factors and ROS generation from peripheral blood leukocytes (PBLs) subjected to a Th1 stimulus. The binding to IgE of egg allergic patients was determined and the peptides present in the hydrolysates were identified, as a contribution to the characterization of hydrolysates that combine the ability to stimulate T lymphocytes and modulate the immune response with a reduced capacity to bind IgE and, eventually, trigger clinical symptoms.

## Materials and Methods

### Ethics statement

All human samples were obtained with written consent from the donors involved in the study. The Bioethics Committee from the Spanish Higher Council for Scientific Research (CSIC) approved all experiments.

### Enzymatic hydrolysates of egg white proteins

Ovalbumin grade VI (OVA), lysozyme (LYS), ovomucoid (OM) and porcine pepsin (EC 3.4.23.1, 3440 U/mg) were purchased from Sigma-Aldrich (St. Louis, MO, USA). Neutrase (EC 3.4.24.28, ≥ 0.8 U/g) and alcalase (EC 3.4.21.62, 2.4 U/g) were both from Novozymes A/S (Bagsvaerd, Denmark). Their lipopolysaccharide (LPS) level, as quantified by the Pierce LAL Chromogenic Endotoxin Quantitation Kit (Thermo Scientific, Waltham, MA, USA), was <1 EU/mg (LYS, OM and the enzymes), except for OVA (440.6 EU/mg). Therefore, commercial OVA was purified using size exclusion chromatography to 3 EU/mg [21].

Hydrolyses with pepsin were carried out on protein solutions in MilliQ water adjusted to pH 1.5 (5 mg/mL), with 172 U/mg of pepsin at 37°C for 24 h, followed by neutralization to pH 7.0. For hydrolyses with Neutrase and alcalase, proteins dissolved in phosphate buffer pH 7.0 (5 mg/mL) were incubated with 0.025 U/mg of Neutrase or 0.005 U/mg of alcalase at 50°C for 60 min. Inactivation of the enzymes was achieved by heating at 95°C for 15 min. The samples were then centrifuged at 5,000 x *g* for 10 min and, when required, fractionated using high performance ultrafiltration units of 10,000 and 3,000 Da cut-off (Amicon Ultra, Millipore, Eschborn, Germany). Hydrolysates and fractions were stored at -20°C prior to subsequent analyses.

The protein content of the hydrolysates and their fractions was determined using the bicinchoninic acid assay (Pierce BCA Protein Assay Kit, Thermo Scientific) and the resulting values were used to standardize their concentration for further experiments.

### RP-HPLC-MS/MS

For RP-HPLC analyses with UV detection (214 nm) and on-line electrospray ionization (ESI-MS/MS), an Agilent 1100 Series HPLC (Agilent Technologies, Waldbronn, Germany) and an Esquire 3000 mass spectrometer (Bruker Daltonik, Bremen, Germany) were used. To aid the identification of disulphide linked fragments, the hydrolysates were analyzed after a reducing step using dithiothreitol, at a final concentration of 70 mM and pH 7.0, for 1 h at 37°C [22].

Chromatographic separations were performed with a RP318 column (250 x 4.6 mm, Bio-Rad). The operating conditions were: flow rate, 0.8 mL/min; injection volume, 50 μl; solvent A, 0.37 mL/L trifluoroacetic acid in Milli-Q water; and solvent B, 0.27 mL/L trifluoroacetic acid in HPLC grade acetonitrile. Elution was conducted with a linear gradient of solvent B in A from 0 to 70% in 75 min, followed by 100% B for 30 min. Ion source parameters were: nebulizer pressure, 60 psi; dry gas, 12 L/min and dry temperature, 350°C. Using Data Analyses TM (version 3.0; Bruker Daltonik), the m/z spectral data were processed and transformed to spectra representing mass values. Biotools (version 2.1; Bruker Daltonik) was used to process the MS(n) spectra and to perform peptide sequencing.

### Human IgE binding by inhibition ELISA

Human IgE-binding of the samples was assessed by inhibition ELISA as previously reported [23], using a pool of 8 different sera from egg allergic children with IgE specific to OVA, LYS and OM ranging between 35.2–1326.5 kU/L, 2.3–36.6 kU/L and 19.3–120.0 kU/L, respectively. Briefly, OVA, LYS and OM were used as coating antigens (0.5, 2.5 and 1.75 μg, respectively). Serial dilutions of the hydrolysates were incubated with the pooled sera (1:1, v/v) and, after 2 h, added to the appropriate coated plate. Polyclonal rabbit anti-human IgE (Dako, Glostrup, Denmark) and polyclonal swine anti-rabbit immunoglobulin labelled with horseradish peroxidase (HRP) (Dako), diluted 1:1000 and 1:2000 (v/v), respectively, were used for detection. A tyramide-biotin and streptavidin-HRP amplification system was employed, following the instructions of the manufacturer (ELAST ELISA amplification system, Perkin-Elmer Life Sciences, Waltham, MA, USA). The reactions were developed using TMB as substrate, stopped with 0.5 M sulfuric acid and the absorbance read at 450 nm in a plate reader (Multiskan FC, Thermo Scientific).

### Th2-primed human PBMCs

Th2-skewed PBMCs were obtained as previously described by Holvoet et al. [24]. Human blood from 8 healthy donors was sterilely collected into ethylenediaminetetraacetic acid tubes. PBMCs were isolated by density gradient centrifugation on Ficoll-Histopaque (GE healthcare, Freiburg, Germany) and suspended in IMDM medium supplemented with 10% fetal bovine serum, 1% L-glutamine, 1% penicillin/streptomycin and 0.1% gentamicin (all from Biowest SAS, Nuaillé, France) at a cellular density of 1.5x10^6^ cells/mL in 48-well plates. PBMCs were stimulated with 50 U/mL of IL-4 (Peprotech, Hamburg, Germany) and 1μg/mL of anti-CD40 antibody (eBioscience, San Diego, CA, USA) to induce a Th2 phenotype. After 3 days in a 5% CO_2_ incubator at 37°C, the intact proteins and their hydrolysates were added at different concentrations (20, 100 and 200 μg/mL) and cultured for a further 3 days in triplicate. As positive control, LPS (1μg/mL, Sigma-Aldrich) was used. Inactivated enzymes were also tested at a concentration equivalent to that present in 200 μg/mL of the hydrolysates. Following incubation, the supernatants were collected and stored at -80°C until their analysis, which was conducted in duplicate.

### Th1-primed human PBLs

PBLs were isolated from human blood from 10 healthy donors and Th1-skewed as described by Richard et al. [25]. Blood was mixed with one volume of 3% (w/v) dextran (Sigma-Aldrich) in 0.9% (w/v) NaCl solution. After 30 min of incubation, PBLs were recovered from the upper layer, erythrocytes were lysed in 0.2% (w/v) NaCl for 30 s, and isotonic osmolarity was re-established by adding one volume of 1.6% (w/v) NaCl. Isolated PBLs were cultured in RPMI 1640 medium supplemented with 0.25% of fetal bovine serum, 1% L-glutamine and 0.5% penicillin/streptomycin (all from Biowest SAS) at a cellular density of 1x10^6^ cells/mL in 48-well plates. PBLs were stimulated with 100 ng/mL of LPS (Sigma-Aldrich) and 20 U/mL of IFN-γ (Peprotech) for 24 h. The intact proteins, their hydrolysates and low molecular mass fractions, were added together with LPS/IFN-γ at a concentration of 200 μg/mL in triplicate. Inactivated enzymes were also tested as explained above. The supernatants were collected and stored at -80°C until their analysis, which was conducted in duplicate.

### Cytokines and IgE

IL-13, IL-5, IL-10, IL-8, IFN-γ and TNF-α were quantified in the supernatants of the corresponding cell cultures using ELISA kits according to the manufacturer’s instructions (eBioscience).

The levels of IgE in the supernatants of Th2-skewed PBMCs were determined by ELISA. 96-well plates were coated with polyclonal rabbit anti-human IgE (Dako) and, after an overnight incubation at 4°C, plates were blocked and incubated at 4°C with the culture supernatants for 12 h. Biotinylated mouse anti-human IgE (Dako) was then added, followed by avidin-HRP (BD Biosciences, San José, CA, USA). The reactions were developed using ABTS as substrate (Roche, Mannheim, Germany) and read at 405 nm.

### Reactive oxygen species (ROS)

Intracellular ROS formation was assessed using the dichloro-dihydro-fluorescein diacetate (DCFH-DA) assay. PBLs cultured with LPS/IFN-γ and 200 μg/mL of the intact proteins, their hydrolysates or low molecular mass fractions for 24 h were labelled with 2.5 μM DCFH-DA in Hanks balanced salt solution (Biowest SAS) and incubated in the dark for 45 min at 37°C [26]. Inactivated enzymes were also assayed. Cells were then washed twice with PBS and subjected to chemical oxidative stress with 0.5 mM *tert*-butyl hydroperoxide (*t*-BOOH) (Sigma-Aldrich) for 15 min. Fluorescence emission due to oxidation of DCFH-DA by intracellular ROS was read at excitation and emission wavelengths of 485 nm and 528 nm, respectively, in a Polarstar Galaxy plate reader (BMG Labtechnologies GmbH, Offenburg, Germany).

### Cell viability

Cytotoxicity of the hydrolysates was determined on cell cultures using 3-[4,5-dimethylthiazol-2-yl]-2-,5-diphenyltetrazoliumbromide (MTT) [27]. Th2-primed PBMCs and Th1-primed PBLs were cultured with 200 μg/mL of the samples in 96-well plates, as described above, followed by the addition of 150 μL of 1 mg/mL MTT (Sigma-Aldrich) to each well and incubation for a further 3 h. The plates were centrifuged at 500 x *g* for 10 min and the supernatants discarded. The formazan precipitate was dissolved by the addition of 50 μL of dimethyl sulfoxide and the absorbance of each well was measured at 570 nm in a plate reader (Multiskan FC, Thermo Scientific).

### Statistical analysis

Data were subjected to ANOVA followed by Dunnett’s *post hoc* multiple-comparison test using the GraphPad Prism package (Version 5; GraphPad, San Diego, CA, USA). In all cases, *P* values< 0.05 were considered statistically significant.

## Results

### The hydrolysates of egg white proteins with pepsin and alcalase inhibit the release of Th2 cytokines and IgE from Th2-primed PBMCs

PBMCs form 6 non-allergic donors, previously selected from a total of 8 volunteers on the basis of a positive induction of Th2 cytokines (IL-5 and IL-13) with anti-CD40 and IL-4, released IL-13 (187.05 ± 53.42 pg/mL), IL-5 (78.16 ± 19.24 pg/mL), IFN-γ (125.41 ± 25.63 pg/mL) and IL-10 (69.36 ± 10.21 pg/mL), following culture with these molecules for 3 days. Levels of IL-13, IL-5 and IL-10 were significantly (*P*< 0.05) higher than those corresponding to the cells cultured with IMDM alone (60.95 ± 29.17, 7.55 ± 4.50 and 17.32 ± 10.87 pg/mL, respectively), while those of IFN-γ were significantly lower (IMDM: 296.80 ± 24.84 pg/mL), thus reflecting a shift to an augmented Th2/Th1 cytokine profile. In view of the variability of the results, the cytokine levels were normalized to those induced by anti-CD40 and IL-4 in each of the 6 selected donors (100%).

**Fig 1:**
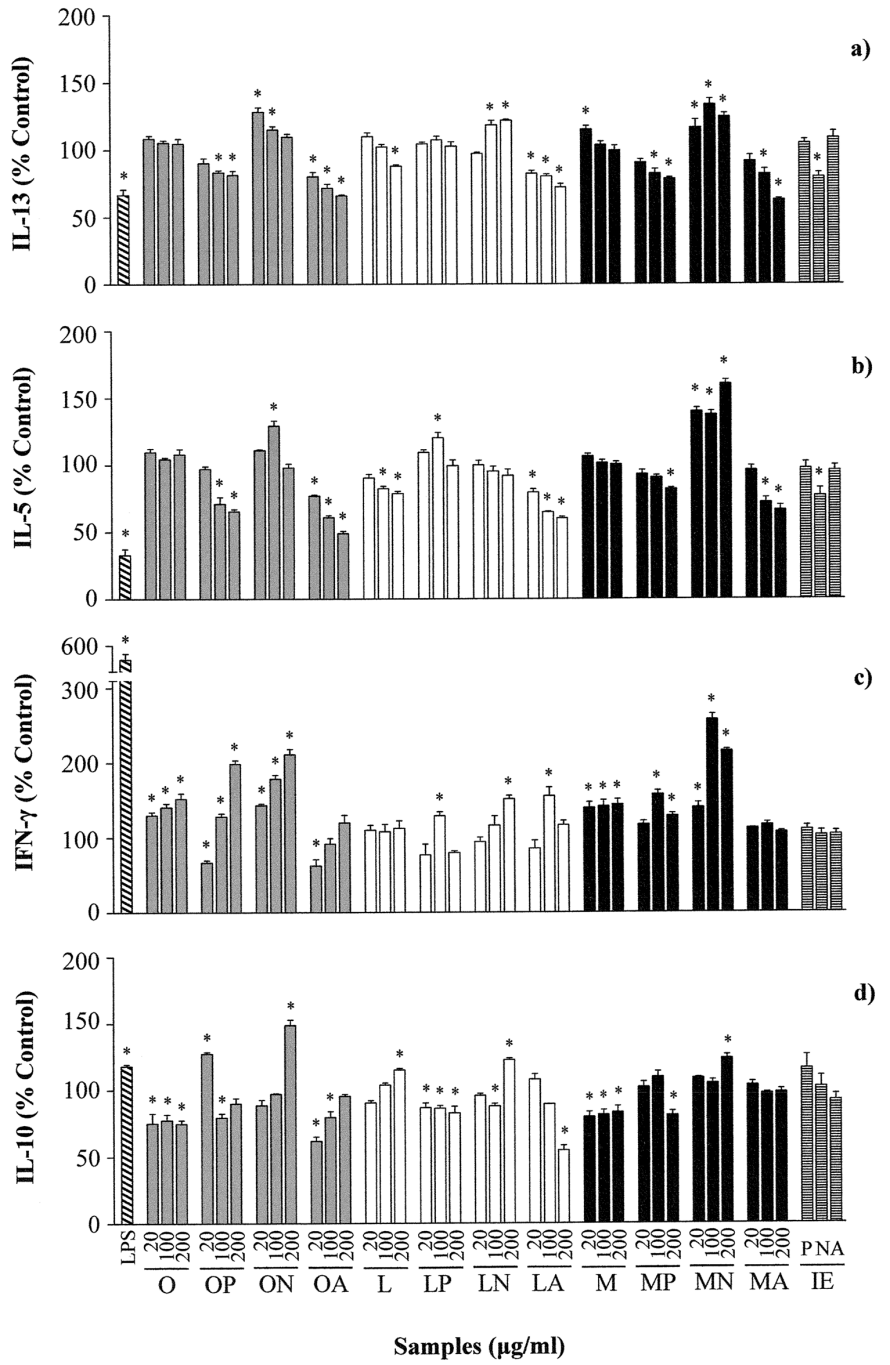
Effects of different concentrations (20, 100 and 200 μg/mL) of ovalbumin (O), lysozyme (L), ovomucoid (M), and their hydrolysates with pepsin (P), Neutrase (N) and alcalase (A), as well as the inactivated enzymes (IE) at a concentration equivalent to that present in 200 μg/mL of the hydrolysates, on the secretion of IL-13 (a), IL-5 (b), IFN-γ (c) and IL-10 (d) by Th2-skewed human peripheral blood mononuclear cells. Data are expressed as percentage of the values induced by anti-CD40 and IL-4 ± standard error of the mean in 6 donors stimulated in triplicate and * indicates significant differences (*P*< 0.05).

As shown in Fig 1, stimulation with LPS significantly down regulated the production of Th2 cytokines (IL-13 and IL-5), and up regulated the release of the Th1 cytokine IFN-γ and that of IL-10.

The hydrolysates of OVA and OM with pepsin and alcalase exerted a dose-dependent inhibition of the release of IL-13 and IL-5 induced by previous anti-CD40 and IL-4 conditioning, which was significant, particularly at the highest doses (Fig 1a and 1b). Likewise, stimulation with the hydrolysate of LYS with alcalase significantly reduced the levels of IL-13 and IL-5 in a dose-dependent manner, although it should be noted that intact LYS also produced a Th2-inhibitory effect similar to that exerted by this hydrolysate. The hydrolysates of egg white proteins with alcalase did not significantly change the secretion of the Th1 cytokine IFN-γ, except for the hydrolysate of LYS with alcalase at 100 μg/mL; while the hydrolysates of OVA, LYS and OM with pepsin at 100 and 200 μg/mL stimulated the release of IFN-γ (Fig 1c).

Conversely, the hydrolysates of OVA and OM with Neutrase increased IL-13 and IL-5 and that of LYS with Neutrase increased IL-13 (Fig 1a and 1b). In general terms, these hydrolysates also stimulated the production of IFN-γ (Fig 1c) and, therefore, they could be regarded as inducers of both Th2 and Th1 responses. Furthermore, the production of IL-10 was significantly enhanced by the hydrolysates of OVA, LYS and OM with Neutrase at the highest concentration (200 μg/mL).

**Fig 2:**
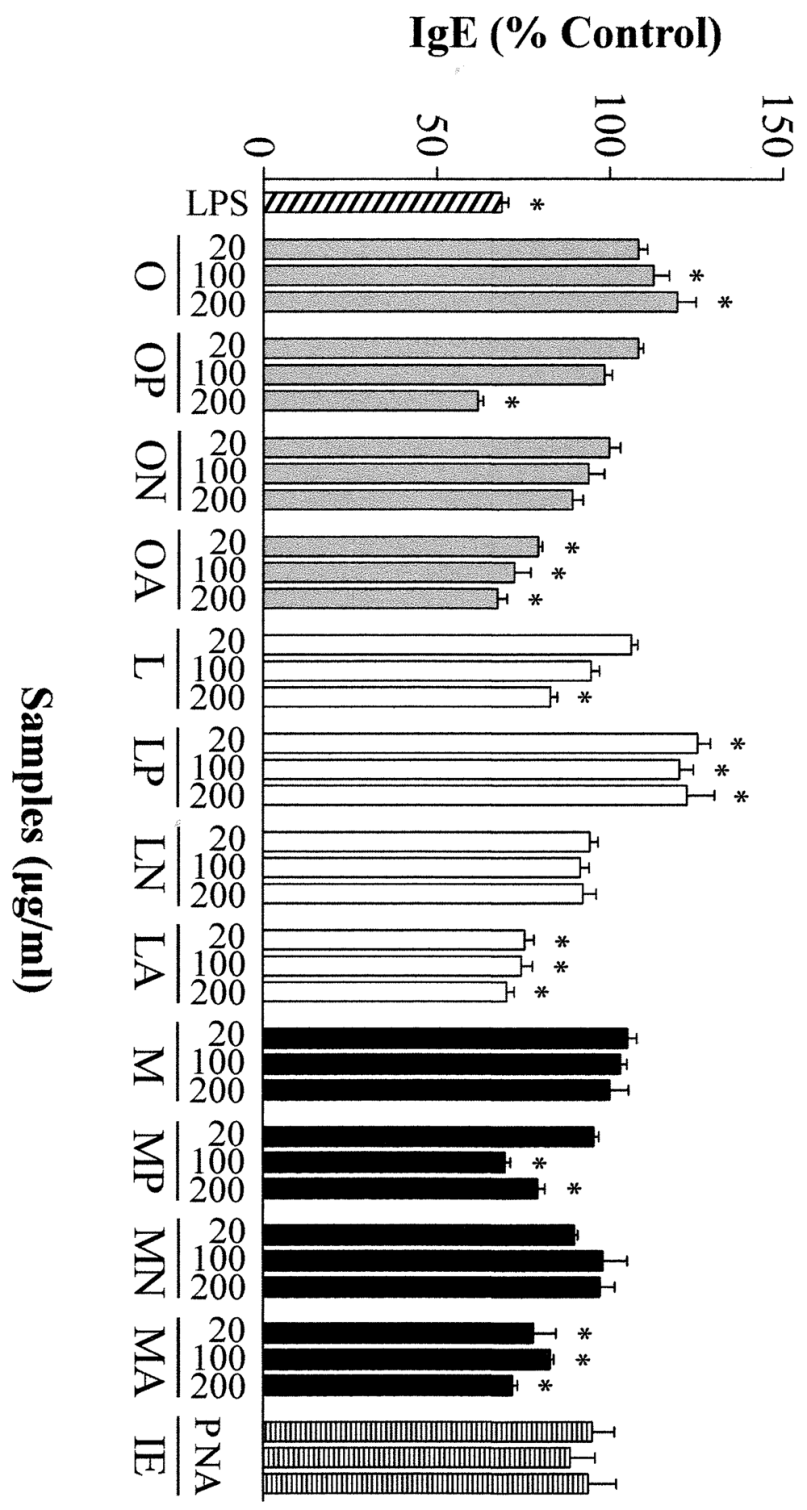
Effects of different concentrations (20, 100 and 200 μg/mL) of ovalbumin (O), lysozyme (L), ovomucoid (M), and their hydrolysates with pepsin (P), Neutrase (N) and alcalase (A), as well as the inactivated enzymes (IE) at a concentration equivalent to that present in 200 μg/mL of the hydrolysates, on the secretion of IgE by Th2-skewed human peripheral blood mononuclear cells. Data are expressed as percentage of the values induced by anti-CD40 and IL-4 ± standard error of the mean in 6 donors stimulated in triplicate and * indicates significant differences (*P*< 0.05).

Conditioning of PBMCs with anti-CD40 and IL-4 induced cells to produce IgE. As shown in Fig 2, incubation with LPS and with the hydrolysates of OVA and OM with pepsin and OVA, LYS and OM with alcalase, that resulted in reduced secretion of IL-5 and IL-13 (Fig 1a and 1b), also inhibited IgE production by primed B cells. Of note is the observation that the hydrolysates of LYS with pepsin enhanced the production of IgE, while those of egg white proteins with Neutrase did not change it, despite, in general terms, they increased Th2 cytokines.

The inactivated enzymes did not have any impact on cytokine or IgE secretion, except for Neutrase, which inhibited IL-13 and IL-5 production by 20 and 33%, respectively (Figs 1 and 2). On the other hand, the MTT assay, carried out in order to assess the cytotoxicity of the stimulation with anti-CD40 and IL-4, as well as that of the proteins and their hydrolysates at a concentration of 200 μg/mL, allowed discarding significant effects on cell viability (Supporting Information, S1 Fig).

### The hydrolysates of egg white proteins with pepsin inhibit the release of pro-inflammatory mediators and the hydrolysates with alcalase hinder ROS generation from Th1-primed PBLs

**Fig 3:**
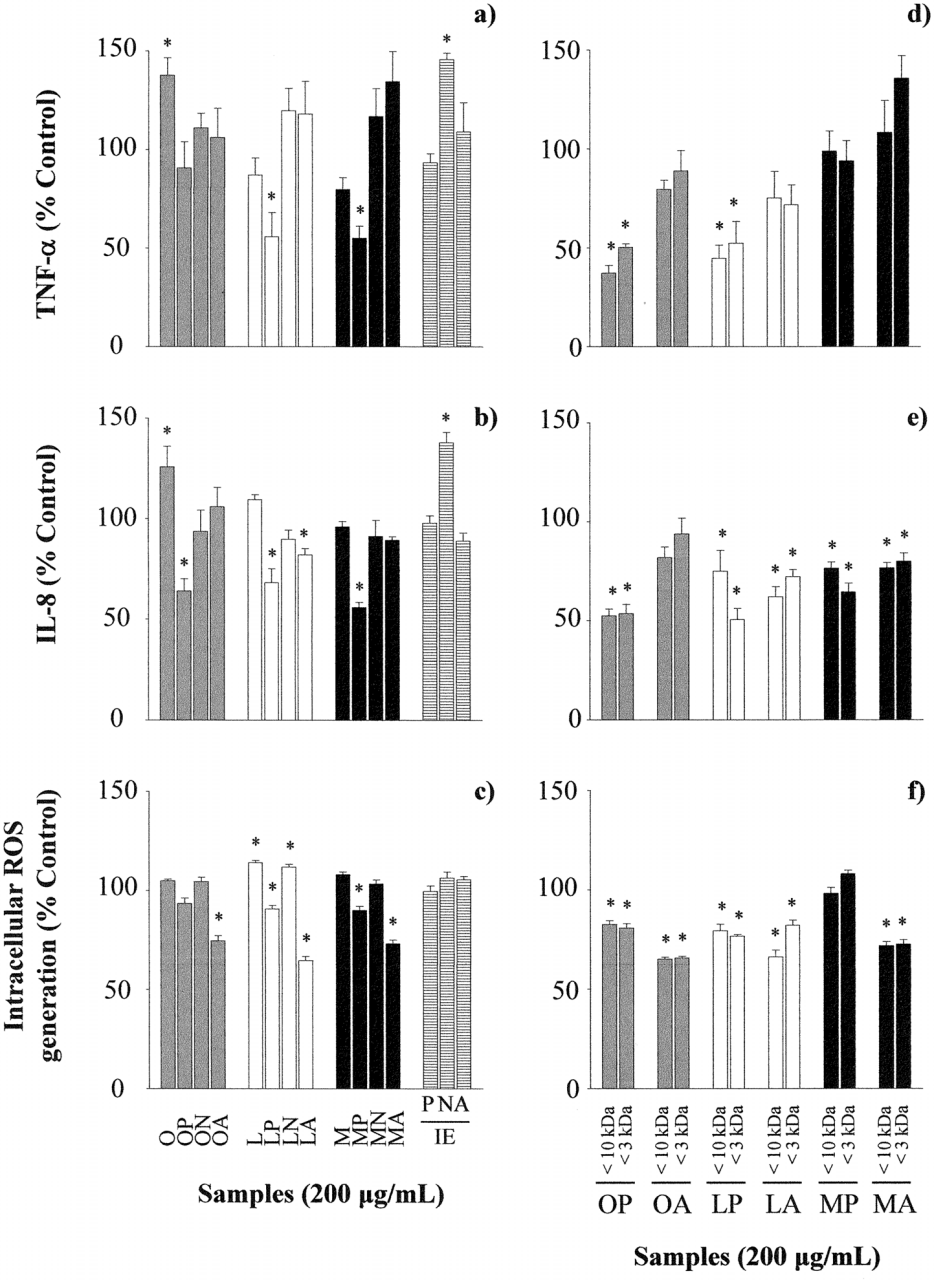
Effects of ovalbumin (O), lysozyme (L), ovomucoid (M), their hydrolysates with pepsin (P), Neutrase (N) and alcalase (A) (200 μg/mL), the inactivated enzymes at a concentration equivalent to that present in 200 μg/mL of the hydrolysates, and the fractions with molecular mass lower than 10,000 and 3,000 Da on the secretion of TNF-α (a, d) and IL-8 (b, e), and intracellular ROS generation (c, f) by Th1-skewed peripheral blood leucocytes. Data are expressed as percentage of the values induced by IFN-γ and LPS ± standard error of the mean in 8 donors stimulated in triplicate and * indicates significant differences (*P*< 0.05).

PBLs from 8 donors were previously selected from a total of 10 volunteers on the basis of a positive induction of an inflammatory response on stimulation with LPS and IFN-γ, which was characterized by the release of mediators such as TNF-α (1048.23 ± 495.12 *vs* 14.59 ± 8.26 pg/mL in RPMI incubated cells, *P*< 0.05) and IL-8 (38467.95 ± 9479.32 *vs* 986.11 ± 321.86 pg/mL in RPMI incubated cells, *P*< 0.05). As shown in Fig 3, OVA increased the production of TNF-α and IL-8, while the hydrolysates of LYS and OM with pepsin reduced the secretion of TNF-α; and those of OVA, LYS and OM with pepsin, and LYS alcalase, reduced the secretion of IL-8 (Fig 3a and 3b). In addition and, in general terms, the hydrolysates of the three proteins with pepsin and alcalase neutralized ROS generation by *t*-BOOH (Fig 3c). Inactivated pepsin and alcalase did not affect the production of cytokines or ROS, but Neutrase significantly stimulated the release of TNF-α and IL-8 (Fig 3). Cytotoxicity assessment with MTT did not reveal any significant adverse effects of LPS or the hydrolysates at the highest concentration (200 μg/mL) on PBLs viability (Supporting Information, S1 Fig).

Since most antioxidant peptides derived from food sources have molecular mass from 500 to 1,800 Da [28], and considering that the best results, in terms of inhibition of the release of pro-inflammatory mediators and oxidative stress in PBLs, were obtained in the cells treated with the hydrolysates of the three egg white proteins with pepsin and alcalase, these were fractionated to assess the contribution of peptides with molecular mass lower than 10,000 and 3,000 Da. The results obtained (Fig 3d, 3e and 3f) suggest that, in general terms, small peptides were responsible for the anti-inflammatory and antioxidant effects observed. The fractions of OVA and LYS with pepsin decreased the secretion of TNF-α by, approximately, 50% (Fig 3d) and, overall, the reduction in the generation of IL-8 and ROS was more marked when cells were incubated with the fractions as compared with the complete hydrolysates (Fig 3e and 3f). However, the fractions derived from the hydrolysates of OM with pepsin exerted a less pronounced effect on all the parameters measured.

### The hydrolysates with pepsin and Neutrase are recognized by human IgE and contain IgE-binding epitopes

We next examined the binding of the hydrolysates to IgE from sera of egg allergic patients (Table 1). The highest IgE-binding corresponded to OVA, followed by LYS and OM. The hydrolysates of OVA, LYS and OM with pepsin maintained, respectively, 10, 41 and 14% of the original IgE-binding and that of OVA with Neutrase, 30%. The IgE binding of the hydrolysates of OVA with alcalase and LYS and OM with Neutrase and alcalase was negligible. RP-HPLC ruled out the existence of intact protein that could account for the residual IgE-binding of the hydrolysates (Supporting Information, S2 Fig).

**Table 1:** IgE binding to sera from egg allergic patients of ovalbumin (O), lysozyme (L), ovomucoid (M) and their hydrolysates with pepsin (P), Neutrase (N) and alcalase (A). Results are means of, at least, 3 independent experiments and are expressed as EC_50_, that is, the effective sample concentration (μg/mL) for 50% of the maximum binding to IgE.

	EC_50_ (μg/mL)
O	2.83 ± 0.23^a^
OP	28.57 ± 0.50^a^
ON	9.43 ± 0.81^a^
OA	2113.5 ± 70.00^b^
L	12.81 ± 2.81^a^
LP	27.80 ± 0.05^a^
LN	1850.00 ± 60.36^b^
LA	851.12 ± 31.32^c^
M	14.62 ± 0.27^a^
MP	193.93 ± 19.73^b^
MN	3070.51 ± 75.85^c^
MA	2295.34 ± 36.95^d^

^a-d^ Different superscripts indicate significant differences (*P*< 0.05) for each protein (O, L or M).

The peptides present in the hydrolysates of OVA, LYS and OM were identified by RP-HPLC-MS/MS (Supporting Information, S3, S4 and S5 Figs). In the hydrolysates of OVA with pepsin, Neutrase and alcalase, 140, 104 and 109 peptides were identified, respectively (Supporting Information, S3 Fig). The hydrolysate of OVA with pepsin only had 11 peptides in common with the hydrolysate with Neutrase, and 12 with the hydrolysate with alcalase, while the hydrolysates with Neutrase and alcalase shared 10 common sequences. Several of the peptides identified in the hydrolysates of OVA with pepsin and Neutrase are comprised within previously described high frequency IgE-binding areas of the protein, such as 125–176, 188–198, 326–336 and 370–385 [29], which could explain their residual IgE-binding. Of note is the observation that the hydrolysate of OVA with pepsin contained peptides with free radical-scavenging properties, such as YRGGLRPINF, YQIGL, FRADHPFL and RADHPFL [OVA (125–134), OVA (212–216), OVA (358–365) and OVA (359–365)] [30]. FRADHPFL was also found the hydrolysate of OVA with Neutrase and RADHPFL in the hydrolysate of OVA with alcalase.

In the hydrolysates of LYS with pepsin, Neutrase and alcalase, we identified 96, 95 and 107 peptides, respectively, that covered the whole LYS sequence, with only 5–8 peptides in common among the different hydrolysates (Supporting Information, S4 Fig). As compared with the hydrolysates with Neutrase and alcalase, the hydrolysate of LYS with pepsin contained larger peptides within the reported IgE-binding areas: 11–27, 57–83 and 108–122 [31]. Furthermore, many of the fragments had sulfhydryl groups, and since RP-HPLC-MS/MS analyses were performed in the presence of a reducing agent to aid identification, it is likely that disulphide-linked fragments contributed to the IgE-binding. Finally, several peptides in the hydrolysate of LYS with alcalase matched sequences previously identified in radical-scavenging fractions, such as KRHGLDNYRGY, KRHGLDNY, HGLDNY, and GLDNYRGY [LYS (13–23), LYS (13–20), LYS (15–20) and LYS (16–23)] [32]. The hydrolysate of LYS with Neutrase contained the defence peptide IVSDGNGMNAW [LYS (98–108)] [33].

In the hydrolysates of OM with pepsin, Neutrase and alcalase, 84, 89 and 86 peptides were identified, respectively, after disulphide bond reduction, with very few common sequences (3–5 peptides) (Supporting Information, S5 Fig). There are numerous IgE-binding epitopes distributed along the OM structure and also many differences in epitope recognition among different egg allergic patients [34]. The observation that, comparatively, longer peptides were found in the hydrolysate of OM with pepsin and that some of them could be linked by disulphide bonds indicates that they could give rise to longer IgE-binding sequences and thus, result in a higher recognition by human IgE.

## Discussion

Following a validated non-antigen specific model [24], a Th2-skewed cytokine profile was induced in human PBMCs from non-allergic donors. Incubation with anti-CD40 and IL-4 led to the production of IL-13, IL-5 and IL-10, and reduced the secretion of IFN-γ in most, but not all, of the donors assayed, and at different concentrations. Similarly, Holvoet et al. reported that the levels of IL-5 were highly donor dependent, although, unlike our results, these authors did not find either IFN-γ or IL-10 production after incubation with IMDM or with anti-CD40 and IL-4 [24]. PBMCs conditioned with anti-CD40 and IL-4 not only imitate allergen-specific cells in the cytokine profile, but also in the secretion of IgE antibodies, because anti-CD40 induces the proliferation of resting B cells and IL-4 co-stimulates anti-CD40 B cell-activation and production of IgE [35]. As expected, culture with LPS reduced the secretion of IL-13 and IL-5 and increased that of IFN-γ, while it also inhibited the release of IgE. Therefore, it redirected the Th2 state in this cell model [24].

The hydrolysates of egg white proteins with alcalase effectively down-regulated the production of Th2 cytokines and the secretion of IgE to the culture media of Th2-skewed PBMCs. The hydrolysates with pepsin produced a similar effect, with the exception of the hydrolysate of LYS with pepsin, which did not reduce IL-5 and IL-13 production and enhanced the secretion of IgE. It has been demonstrated that the inhibition of IgE production by plasma cells exerted in vitro by anti-inflammatory substances correlates in vivo (in a mouse model of peanut allergy) with suppression of IgE and decreased adverse reactions and plasma histamine levels [36]. Therefore, protein hydrolysates with the ability to prevent IgE secretion by B cells might impair the availability of this antibody for participation in mass cell activation, offering protection against the development of anaphylactic symptoms.

Our results show that the hydrolysates of OVA, LYS and OM with alcalase combined in vitro neutralization of an excessive Th2 response and reduction of IgE secretion with a low grade cytokine-stimulating role, which may correlate with an anti-allergic protection in vivo due to a homeostatic effect [24]. On the other hand, the hydrolysates of OVA and OM with pepsin helped to re-establish the Th1/Th2 balance in the Th2-skewed cells, as they simultaneously decreased the production of IL-5 and IL-13 and increased that of IFN-γ. It should be noted that both the inhibition of allergen-induced Th1 and Th2 cytokine responses [17], as well as the promotion of Th1-biased responses to a detriment of Th2 ones [18], have been associated to the therapeutic effects of egg white peptides in mouse models of allergy.

We next evaluated the anti-inflammatory and antioxidant properties of the hydrolysates on cells of the peripheral blood. For this purpose, we used PBLs, that contain, in addition to lymphocytes and monocytes (PBMCs), polymorphonuclear cells, which participate, together with macrophages, in the initiation and progression of inflammatory processes once being recruited to the required sites [5]. In order to induce inflammatory responses, PBLs were stimulated with LPS and IFN-γ, which, in agreement with previous results, led to the production of TNF-α and IL-8 [25]. The hydrolysates of LYS and OM with pepsin inhibited the release of TNF-α and IL-8 in PBLs treated with inflammatory stimuli and also reduced oxidative stress in cells in which ROS generation was induced by *t*-BOOH treatment. This suggests their potential to counteract the inflammatory damage that may arise in response to allergic injuries. In turn, the hydrolysates of OVA, LYS and OM with alcalase did not avoid the release of pro-inflammatory mediators, but significantly neutralized the oxidative stress induced by *t*-BOOH. It is likely that, in these hydrolysates, except in that of OM with pepsin, peptides with molecular mass lower than 10,000 Da were responsible for the anti-inflammatory and antioxidant effects observed. Pepsin digestion of OM gives rise to large fragments linked by disulphide bonds [37] (as shown in Supporting Information, S2 Fig) and, therefore, the content of free low molecular mass peptides in this hydrolysate is probably small.

Due to the cross-regulatory interaction between Th1 and Th2 immunity, it is hypothesized that the suppression of Th1 responses by antioxidant compounds, while beneficial in reducing the side effects of the inflammatory processes, may lead to an up-regulation of Th2-type cytokines that promotes allergic sensitization and exacerbates allergic symptoms [6]. However, in our experiments, we did not observe an inverse regulation of cytokine production in the Th2- and Th1-skewed cell models. The hydrolysates of OVA and OM with pepsin and the hydrolysates of OVA, LYS and OM with alcalase concurrently down-regulated features characteristic of exacerbated Th2 and Th1 responses, which suggests their potential for allergy treatment. Conversely, the hydrolysates of OVA, LYS and OM with Neutrase tended to promote both Th2 and Th1 activation in the Th2-skewed model and they could not attenuate the induction of inflammatory mediators in the Th1-skewed model. In any case, it cannot be excluded that the heat-inactivated Neutrase preparation exerted an immunomodulating effect that would have biased the influence of the egg white protein derived peptides, particularly on the Th1-primed cells.

The cell models used in the present work allowed the estimation of the influence of the hydrolysates on the cytokine profiles that modulate allergic responses with the use of accessible human cells in in vitro tests. Probably because of the low frequency of allergen-specific T cells, previous attempts to assess the immunomodulatory effects of egg proteins on PBMCs from egg allergic patients were unsuccessful due to the lack of sufficient stimulation leading to cytokine and antibody secretion (results not shown). In this respect, it should be noted that, although allergen-specific T cell lines have been used to overcome the frequency limitation, in vitro expansion can alter cell phenotypes or bias the results through the selection of the rapidly proliferating clones [38].

IgE-binding was comparatively higher in the hydrolysates with pepsin (as well as in the hydrolysate of OVA with Neutrase), which was related to the length of the peptides formed and the existence of disulphide linked fragments that could carry bivalent epitopes. The low IgE-binding of the hydrolysates with alcalase could be attributed to the fact that they contained short peptides, with molecular masses lower than 1500 Da. Pepsin digestion has already been shown to reduce the reactivity of IgE from human sera to egg white proteins, such as OVA [29], LYS [31], and OM [37]. Indeed, pepsin hydrolysis of fish and nut allergens effectively down-regulates the allergic responses they cause in sensitized humans and mice [39, 40]. Our work shows that the hydrolysates of OVA and OM with pepsin modulated Th2 cytokines and IgE secretion, while attenuating inflammatory responses. Comparatively, the hydrolysates of OVA, LYS and OM with alcalase also equilibrated Th2 differentiation efficiently, but exhibited a more reduced IgE-binding. Therefore, it can be hypothesized that these hydrolysates would elicit milder allergic reactions while retaining T cell-stimulating abilities, which might correlate with an anti-allergic benefit.

## Supporting Information

S1 Fig Cytotoxicity of the hydrolysates determined by the MTT method. (PPTX)

S2 Fig RP-HPLC patterns of the hydrolysates of ovalbumin, lysozyme and ovomucoid produced with pepsin, alcalase and Neutrase. (PPTX)

S3 Fig Peptide sequences identified by RP-HPLC-MS/MS in the hydrolysates of ovalbumin with pepsin, Neutrase and alcalase. (PPTX)

S4 Fig Peptide sequences identified by RP-HPLC-MS/MS in the hydrolysates of lysozyme with pepsin, Neutrase and alcalase. (PPTX)

S5 Fig Peptide sequences identified by RP-HPLC-MS/MS in the hydrolysates of ovomucoid with pepsin, Neutrase and alcalase. (PPTX)
