# Supplementary material for: Regulation of Exacerbated Immune Responses in Human Peripheral Blood Cells by Hydrolysed Egg White Proteins
Source: PLoS One. 2016 Mar 23;11(3):e0151813. doi: 10.1371/journal.pone.0151813 (PMC4805267; doi:10.1371/journal.pone.0151813)
Supplement: S1 Fig — (PPTX) [file pone.0151813.s001.pptx]

## Slide 1
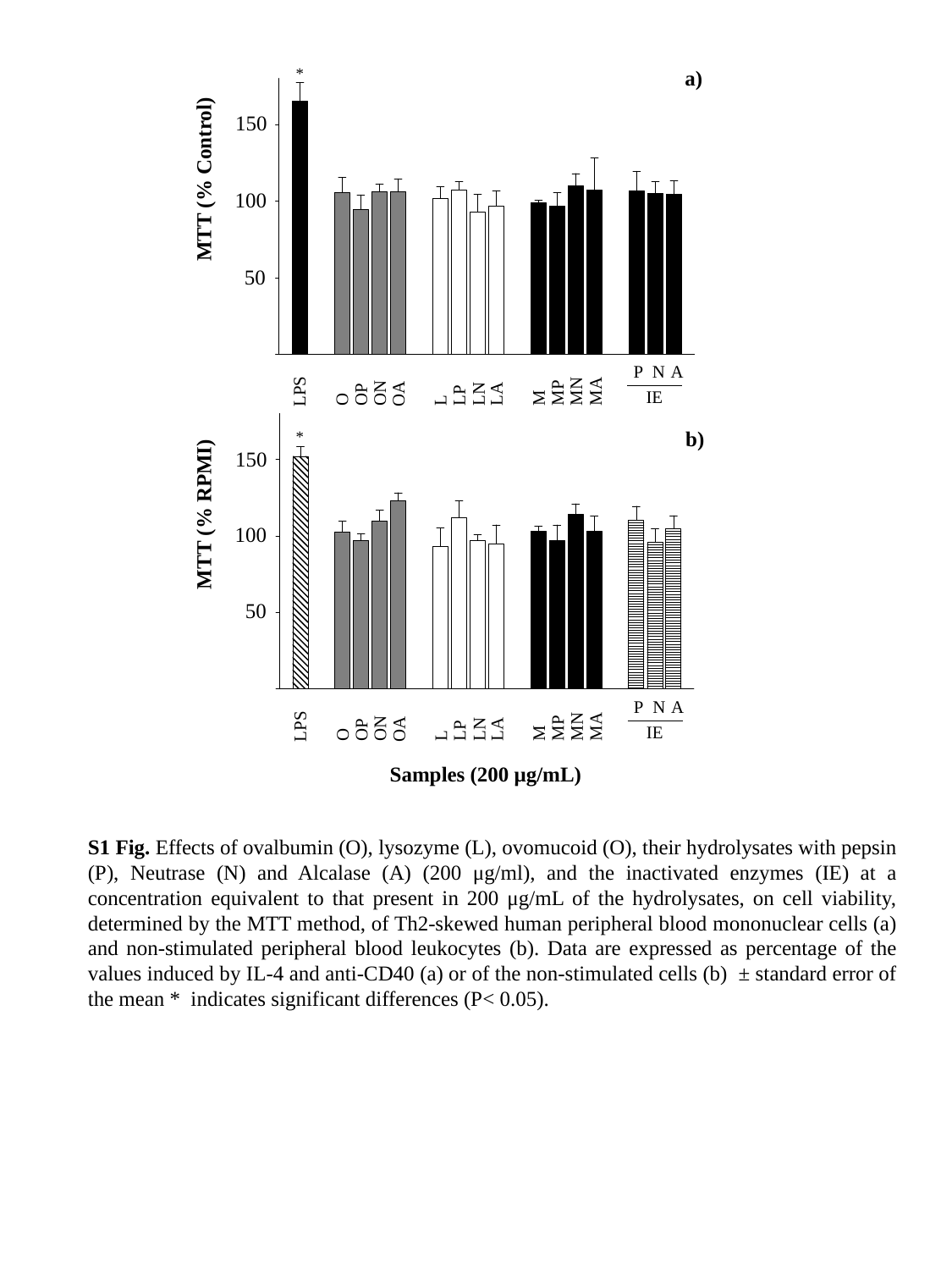

*
a)
150
MTT (% Control)
100
50
P
N
A
IE
LPS
M
MN
MA
MP
L
LN
LA
LP
O
ON
OP
OA
b)
*
150
MTT (% RPMI)
100
50
P
N
A
IE
LPS
M
MN
MA
MP
L
LN
LA
LP
O
ON
OP
OA
Samples (200 μg/mL)
S1 Fig. Effects of ovalbumin (O), lysozyme (L), ovomucoid (O), their hydrolysates with pepsin (P), Neutrase (N) and Alcalase (A) (200 μg/ml), and the inactivated enzymes (IE) at a concentration equivalent to that present in 200 μg/mL of the hydrolysates, on cell viability, determined by the MTT method, of Th2-skewed human peripheral blood mononuclear cells (a) and non-stimulated peripheral blood leukocytes (b). Data are expressed as percentage of the values induced by IL-4 and anti-CD40 (a) or of the non-stimulated cells (b) ± standard error of the mean * indicates significant differences (P< 0.05).
