# Supplementary material for: Regulation of Exacerbated Immune Responses in Human Peripheral Blood Cells by Hydrolysed Egg White Proteins
Source: PLoS One. 2016 Mar 23;11(3):e0151813. doi: 10.1371/journal.pone.0151813 (PMC4805267; doi:10.1371/journal.pone.0151813)
Supplement: S2 Fig — (PPTX) [file pone.0151813.s002.pptx]

## Slide 1
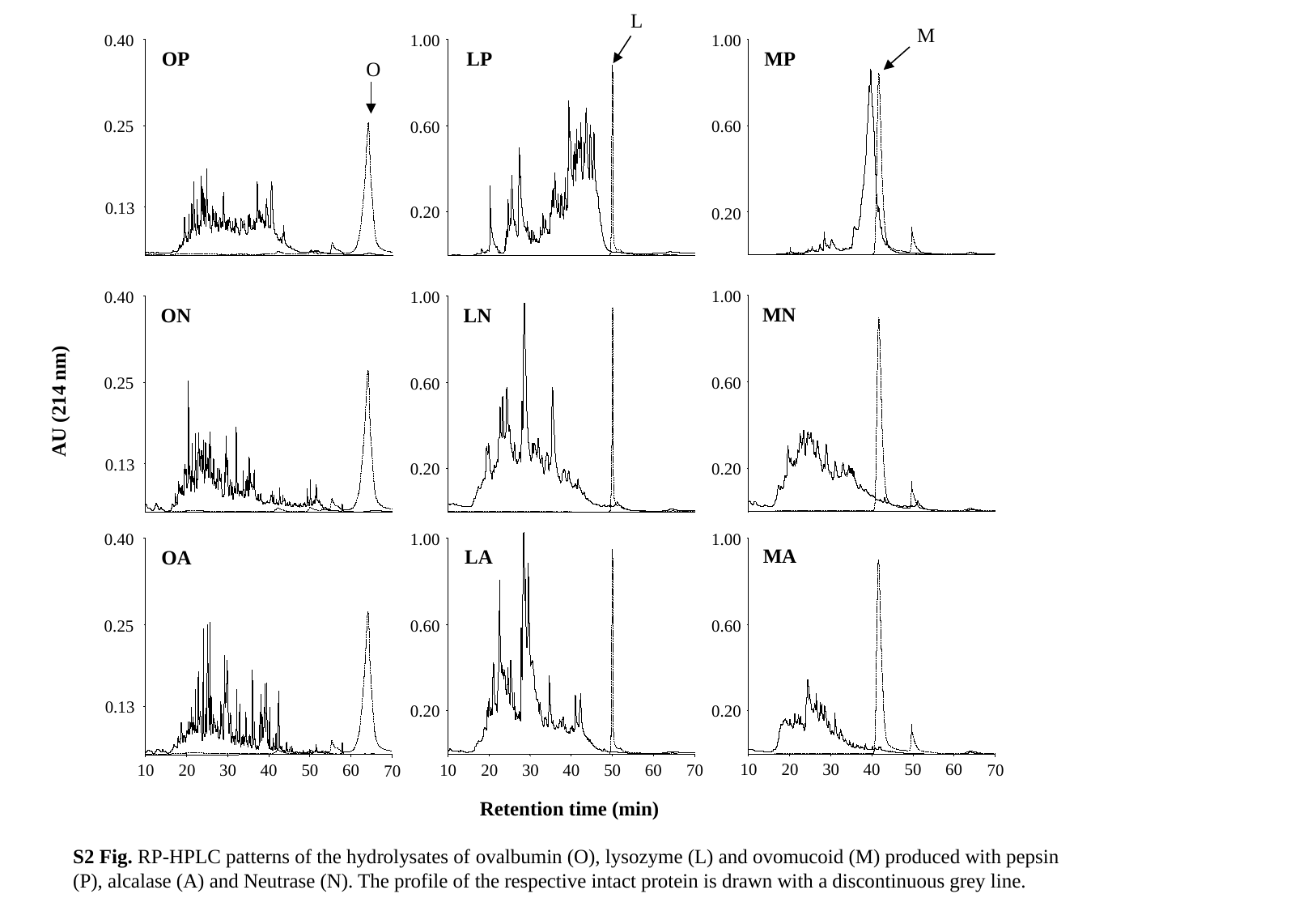

L
M
1.00
1.00
0.40
LP
OP
MP
O
0.25
0.60
0.60
0.13
0.20
0.20
1.00
1.00
0.40
MN
ON
LN
0.60
0.25
0.60
0.13
0.20
0.20
1.00
1.00
0.40
MA
LA
OA
0.25
0.60
0.60
0.13
0.20
0.20
10
20
30
40
50
60
10
20
30
40
50
60
70
10
20
30
40
50
60
70
70
AU (214 nm)
Retention time (min)
S2 Fig. RP-HPLC patterns of the hydrolysates of ovalbumin (O), lysozyme (L) and ovomucoid (M) produced with pepsin (P), alcalase (A) and Neutrase (N). The profile of the respective intact protein is drawn with a discontinuous grey line.
